# Quantitative assessment and comparison of susceptibility to colibacillosis in pure lines of broiler breeders and their commercial offspring

**DOI:** 10.1016/j.psj.2025.105722

**Published:** 2025-08-24

**Authors:** T.T.M. Manders, A. Papanikolaou, J.J. de Louwere, M.G.R. Matthijs

**Affiliations:** Department of Population Health Sciences, Faculty of Veterinary Medicine, Utrecht University, Utrecht, the Netherlands

**Keywords:** *E. coli*, Colibacillosis, Genotype, Meat-type chicken, Susceptibility

## Abstract

Colibacillosis, caused by avian pathogenic *Escherichia coli* (APEC), is a disease of major economic importance to the broiler industry. This study aimed to investigate genetic variation in susceptibility to colibacillosis by comparing four pure broiler breeder lines and their commercial four-way cross offspring. Three consecutive experiments were performed assessing mortality, growth retardation and mean lesion scores (MLS) after *E. coli* challenges. In the first experiment, birds were challenged intratracheally with a high dose of *E. coli* at 8 days of age. All pure lines showed significantly higher mortality rates (54.2-80.6 %) compared to the commercial line (20.9 %).

The second experiment tested the most and least susceptible pure lines from Experiment 1, alongside the commercial line. Low, medium, and high *E. coli* doses were inoculated at the same inoculation day as Experiment 1. A clear dose-dependent effect on mortality and growth was observed across all lines, confirming that disease severity is linked to challenge dose.

The third experiment compared the most susceptible pure line and the commercial line using a dual-infection model. Prior to the *E. coli*-inoculation, an infectious bronchitis virus (IBV) vaccine strain was administered. The results were in line with the first two experiments, the commercial line performed better on all parameters compared to the tested pure line. No significant differences in susceptibility were found between males and females in any experiment.

The commercial broilers, a four-way cross of the tested pure lines, demonstrated superior resistance to colibacillosis compared to the pure lines in all experiments. Among the pure lines, genetic differences in susceptibility to colibacillosis were observed. These differences indicate that genetic selection for resistance to colibacillosis might be feasible.

## Introduction

Colibacillosis is a disease of great economic importance for the broiler industry due to its variable, but potentially high mortality rates (Dho-Moulin and Fairbrother, 1999; Nolan et al., 2020), its adverse effects on growth (Goren, 1991; Russell, 2003) and due to carcass condemnation at slaughter (Haslam, et al., 2008; Yogaratnam, 1995). Colibacillosis in chickens refers to any localized or systemic infection caused by avian pathogenic *Escherichia coli* (APEC). Clinical signs include stunted growth, lameness, unresponsiveness, lethargy and moribundity. However, these signs can vary, being fewer and milder in localized infections compared to systemic infections (Nolan, et al., 2020).

In broilers, infections with APEC can occur as single *E. coli* infection, which typically arise without any specific triggers and are most common during the first 14 days of life. After 14 days of age, however, colibacillosis usually require a trigger to develop. A common example of such a trigger is an infection with a respiratory virus, such as infectious bronchitis virus (IBV) (Matthijs, et al., 2009; Nakamura, et al., 1992).

Antimicrobial drugs are still effectively used to mitigate losses caused by colibacillosis. However, growing concerns about antibiotic resistance have driven efforts to explore alternative approaches. Various strategies to reduce the clinical impact of *E. coli* infections in broilers have been investigated, including the supplementation of biotics, use of bacteriophages, biosecurity measures, vaccination, acidifiers, and phytochemicals. In most studies, the supplementation of prebiotic, probiotic, and symbiotic products did not significantly affect the occurrence of colibacillosis (Li, et al., 2021; Peek, et al., 2013; Tarabees, et al., 2019). Other studies have extensively investigated the application of bacteriophages against APECs (Huff, et al., 2002; 2005; 2010). However, limitations such as narrow host specificity of the phages, the timing of administration, and the development of resistance to phages have rendered this approach impractical for large-scale application in the poultry industry.

Another possible alternative to the use of antibiotics might be genetic selection for disease resistance. In poultry, successful selection for resistance to several diseases, such as Marek’s disease, avian influenza, and Newcastle disease, has been demonstrated (Drobik-Czwarno, et al., 2018; Francis and Kish, 1955; Idoko-Akoh, et al., 2023; Schat, et al., 1994). If significant differences in susceptibility to colibacillosis exist between chicken lines, it may be possible to develop specific chicken lines through genetic selection that are less susceptible to colibacillosis or are better able to cope with the disease. Results of the genetic selection could be a reduced impact of colibacillosis in commercial broilers and improve colibacillosis prevention and control. There is evidence that such genetic variation in susceptibility to colibacillosis exists (Ask, et al., 2006; Calenge, et al., 2014; Monson and Lamont, 2021). However, none of these studies assessed the susceptibility to APEC in pure lines of meat type-chickens, or compared them with the four-way cross offspring of the tested pure lines.

Two animal infection models have been has developed to study the pathogenesis of colibacillosis and test interventions to prevent or treat colibacillosis (Berghof, et al., 2019b; Dwars, et al., 2009; Matthijs, et al., 2003; Wijnen, et al., 2022), These infection models (single and dual) are highly reproducible, and outcome parameters used, such as mortality rates and the amount of purulent exudate observed during post-mortem examination, expressed as the Mean Lesion Score (MLS), are of practical relevance.

In the single-infection model, broiler chickens are intratracheally inoculated with *E. coli* (O78K80) (van Eck and Goren, 1991) at 7 or 8 days of age. In the dual-infection model, chickens are first inoculated with infectious bronchitis virus (IBV) at approximately 3 weeks of age via ocular, intranasal, and/or intratracheal routes, followed by intratracheal inoculation with the same *E. coli* as the single-infection model five days later.

The aim of this study was to investigate whether there are differences in susceptibility to colibacillosis between four pure lines of broiler breeders and commercial broilers. Additionally, differences in susceptibility between males and females within the same chicken line were assessed. The commercial broilers were the result of a four-way cross of the tested pure lines. To address this question three animal experiments were performed. In the first experiment, four different pure lines of broiler breeders and commercial broilers were tested using the single *E. coli* infection model. Based on the results of Experiment 1, the most and less susceptible base pure broiler line were selected for the second animal experiment and compared to the commercial broilers. These lines were tested with three different *E. coli* concentrations—high, medium, and low—to determine if genetic susceptibility to colibacillosis is dose-dependent. In the last animal experiment the most susceptible pure line and the commercial line were compared in the dual infection model (IBV and *E. coli*). In all experiments, the mortality rate, growth retardation and postmortem mean lesion scores were assessed after the *E. coli* challenge.

## Material and methods

### Ethical statement

All animal experiments have been approved by the Animal Welfare Committee (DEC); under license number 2014.II.09.073 and by the Animal Welfare Body (IVD) of Utrecht University. The experiments are in compliance with the Dutch legislation and the EU Directive on the protection of animals used for scientific purposes (2010/63/EU).

### Experimental designs and rationale

**Fig. 1:**
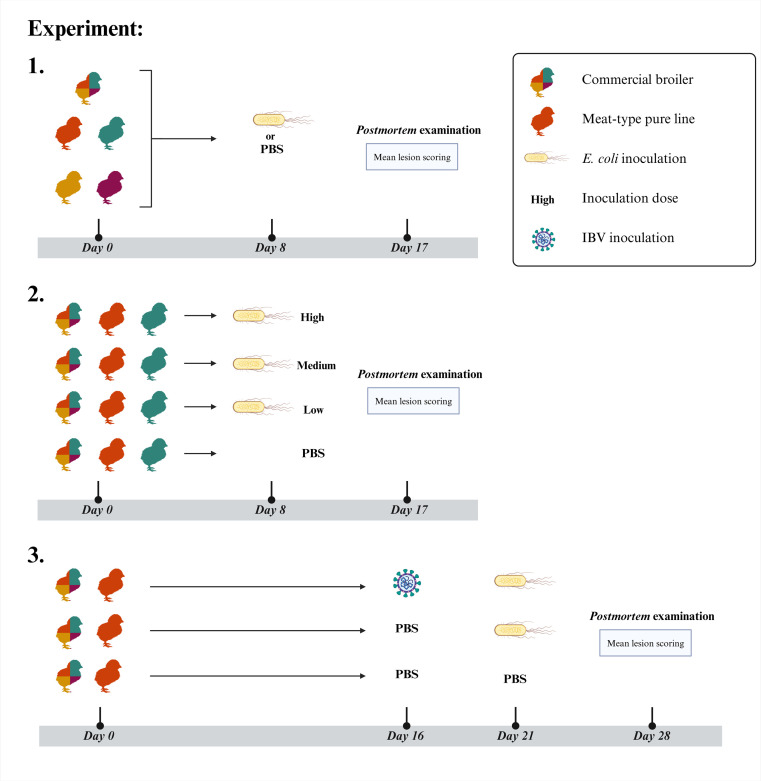
Schematic overview of the experimental design. Three experiments were conducted. In Experiment 1, four pure-line meat-type chickens were intratracheally inoculated with *Escherichia coli* (10^7.1^ colony forming unit (CFU)/ bird) at day 8 of age (further referred to as day 8). The most and least susceptible pure lines were exposed to three different doses of *E. coli* at day 8 in experiment 2, doses used were: 10^7.1^, 10^6.1^, 10^5.1^ CFU per bird and classified as high, medium and low, respectively. In experiment 3, a dual-infection model was used: birds were inoculated with infectious bronchitis virus (IBV) H52 at day 16 and five days (day 21) later with *E. coli* (10^7.0^ CFU/ bird). Commercial broilers, resulting from a four-way cross of the tested pure lines, were included in all three experiments and used as references groups. For each pure-line and commercial broiler group, a mock-inoculated group was included in all experiments, these birds were inoculated with phosphate-buffered saline (PBS). Created in https://BioRender.com.

In total, three consecutive experiments were conducted (Fig. 1). The susceptibility to colibacillosis of four different pure line of broiler breeders and commercial broilers, resulting from a four-way cross of the tested pure lines, were assessed using a single *E. coli* infection model (Experiment 1). In this model, birds were inoculated on day 8 of age (further referred to as day 8), either with a high dose of *E. coli* (10^7.2^ CFU/bird) or with phosphate-buffered saline (PBS). The experiment was terminated on day 17. The PBS-inoculated groups served as controls, resulting in ten groups of chickens in Experiment 1.

In previous studies using the same *E. coli* infection model, dose-dependent differences were observed in commercial broilers (Matthijs, et al., 2003; Velkers, et al., 2005). To determine whether genetic susceptibility to colibacillosis is dose-dependent, the pure lines with the highest and lowest mortality in Experiment 1 (lines A and B, respectively), along with the commercial line, were selected for the second experiment. The same single-infection model was used; however, three different doses of *E. coli,* low, medium, and high (10^5.1^, 10^6.1^, and 10^7.1^ CFU/bird), or PBS were inoculated. A total of twelve groups of chickens were included in this experiment. The inoculation day and the endpoint of Experiment 2 were the same as in Experiment 1, *i.e.*, day 8 and day 17, respectively.

In the final animal experiment the most susceptible pure line (Line A) and the commercial line were compared in the dual infection model. For each line, three different treatments were applied, resulting in six groups. In the first treatment, an infectious bronchitis vaccine virus was administered on day 16 followed by an *E. coli* challenge on day 21. In the second treatment, PBS was inoculated on day 16 and *E. coli* on day 21. In the control groups, PBS was inoculated at day 16 and 21. The experiment terminated on day 28.

In all three experiments, the growth and mortality of the chickens were recorded throughout the experiments. At the end of each experiment, post-mortem examinations were performed on the euthanized chickens. Based on the growth and mortality parameters in all three experiment the production loss index for each chicken line was calculated (see “Mortality, body weight, production loss index”).

### Animals and husbandry

In all three experiments, eighteen-day-old embryonated eggs originating from parent flocks of the different pure lines of broiler breeders (A, B, C and D) were obtained from the hatchery of the breeder company and eighteen-day-old embryonated eggs originating from different parent flocks of the commercial broiler line and were obtained from commercial hatcheries (Morren B.V., Lunteren, the Netherlands (Experiment 1), Moonen & Wagemans, Nederweert, the Netherlands (Experiment 2 and 3)). The last three days of incubation were completed at the research facility of the Faculty of Veterinary Medicine of Utrecht University.

On the day of hatch, the birds were cloacally sexed, individually labeled, weighed, and divided into two groups with an equal ratio of roosters and hens (Supplementary Table 1). In total 822 birds were inoculated in Experiment 1, of which 133, 188, 141 and 189 birds of the commercial line, pure line A, B, C, and D, respectively. Approximately half of the birds were inoculated with either *E. coli* or PBS. In the second experiment, 809 birds were inoculated in total; of which 281, 266, and 262 of the commercial line, pure line A and B, respectively. The birds were equally divided per line across the treatment groups. In the last experiment, 119 birds were inoculated in total of which 60 of the commercial line and 59 of pure line A. The birds were equally divided across the treatments groups, resulting in 20 birds per group (Supplementary Table 1 to 3). Thereafter, the birds were housed in a research unit with pens measuring 3.0 m². Each pen was provided with 1,500 grams of Lignocel hygienic animal bedding (Experiments 1 and 2). The research unit was ventilated at a rate of approximately 40 m^3^ per hour, and the room temperature was gradually decreased from 28.9°C on day 1 to 25.3°C on day 17. During the first 5 days post hatch, each pen was equipped with a heat lamp. In Experiment 3, birds were housed in negative-pressure isolators (Beyer and Eggelaar, Utrecht, the Netherlands), in order to prevent transmission of infectious bronchitis virus between the group. From the day of hatch, these birds were placed in isolators with a volume of 1.3 m^3^, each fitted with a slatted floor of 1.05 m^2^. The isolators were ventilated at a rate of approximately 30 m^3^ per hour with room temperature of 35°C on day of hatch, which was gradually decreased to 22.5°C by day 23 of age. This temperature was maintained until the end of the experimental period.

In all experiments, birds were fed a commercial broiler feed containing 12.3 MJ/kg metabolizable energy and 19.9 % crude protein. Both, feed and tap water were provided *ad libitum* throughout the experimental period in Experiment 1 and 2. In Experiment 3, commercial feed and tap water were provided *ad libitum* for 14 days. Thereafter, only the feed was restricted on a "skip-a-day" basis to prevent leg disorders and hydrops ascites, which pose a risk when broilers are housed in isolators. Per chicken, the following amount of feed was provided: 127 g at 15 days post hatch (dph), 149 g at 17 dph, 172gr at 19dph, 195 g at 21 dph, 221 g at 23 dph, 246 g at 25 dph and 272 g at the age of 27 days.

In all experiments, continuous light was provided for the first 36 hours post hatch. After this period, a light scheme of 15 hours light and 9 hours dark was implemented in experiment 1 and 2 until the end of the experiments. While in experiment 3, a 16 hours light and 8 hours dark scheme was implemented. In Experiment 3, red light was used from 4 days of age to prevent cannibalism.

### Inoculation strain, method, preparation and dose

#### Escherichia coli

*E. coli* strain 506 has been extensively used in other animal experiments. This strain originated from the pericardium of a broiler with colibacillosis (van Eck and Goren, 1991) and was used as the inoculation strain in all three experiment of this study. Inoculum preparations were performed as described and by Matthijs, et al. (2003). In brief, the *E. coli* was recovered from cryopreservation, plated on sheep blood agar plates and incubated overnight. Colonies were then scrapped from plate with a dry sterile cotton swab and suspended in physiological saline until an optical density of 0.5 McFarland was reached. One ml of this bacterial suspension was diluted in 9 ml 0.5 % glucose broth (Thermo Scientific™, Nutrient broth with glucose, Schwerte, Germany) and incubated for 16 hours at 37°C to obtain a suspension of approximately 5 × 10^8^ cfu/ml. Serial dilutions in PBS were made to reach the desired inoculation dose. The final concentrations of the inocula were assessed by means of bacterial colony count directly after inoculation. The inocula were stored on melting ice at all times, except when being handled. Negative control groups were inoculated with PBS.

In all experiment, the birds were inoculated intratracheally using a 1.0 ml syringe fitted with a blunt-ended pipette tip (catalogue number 4862; Corning, New York, USA). Inoculations were performed with a volume of 0.3 ml, resulting in an inoculation dose of 10⁷^.^² colony-forming units (CFU) per bird in experiment 1, and 10⁷^.^¹ (high dose), 10⁶^.^¹ (medium dose) or 10⁵^.^¹ (low dose) CFU per bird in experiment 2. In the last experiment, 0.5 ml of the inoculum was inoculated intratracheally, resulting in an inoculation dose of 10⁷^.0^ CFU per bird. Birds in the control groups were inoculated with 0.3 ml (Experiment 1 and 2) or 0.5 ml (Experiment 3) of PBS.

### Infectious bronchitis virus

For the infectious bronchitis virus (IBV) inoculation in experiment 3, a vaccine strain (AviPro IB H52, Lohmann Animal Health GmbH, Germany) was used. The vaccine virus was obtained as commercial freeze-dried vials. Prior to inoculation, virus titration of the IBV H52 vaccine was performed in 10-day-old embryonated specific-pathogen-free white leghorn (SPF-WL) eggs as described by de Wit, et al. (1998). The IBV inoculum contained 10⁵^.^⁷ EID₅₀/ml of IBV H52. Each bird received 0.05 ml in each eye and nostril and 0.5 ml intratracheally, resulting in a total IBV inoculum volume 0.7 ml per bird.

### Mortality, body weight, production loss index

Body weights of all birds during the experiments were assessed, all chickens were weighted on days 1, 7, 12, 15 and 17 in Experiment 1. In the second experiment on days 1, 7, 11 and 17 and in the third experiment on days 1, 7, 15, 21 and 28. The weighing of the birds was performed with two calibrated balances (Kern EMB2200-0 and Kern FOB 3K1). In experiment 1 and 2, mortality was recorded four times per day and in the third experiment, once a day.

Loss in body weight due to colibacillosis was calculated as an index using a formula described by van Eck and Goren (1991), further referred to as Production Loss Index (PLI).

PLI is based on weight loss in the *E. coli* inoculated group due to growth depression and mortality. Weight loss of live birds in the *E. coli* group is defined as mean body weight of live birds in the placebo group minus mean body weight of live birds in the *E. coli* inoculated group, times the number of life birds in the *E. coli* group. Weight loss due to mortality is defined as mean body weight of live birds in the placebo group times the number of dead birds in the *E. coli* group. If mortality occurred in the placebo group the number of live birds in the *E. coli* group was corrected for the number of dead birds in the placebo group.

In a formula:

$$PLI=\frac{\left(№\;Se\;+\;№\;\text{Dp}\right)\left(\text{Wp}-We\right)\;+\;\left(№\;De-№\;\text{Dp}\right)\;\times \;\text{Wp}}{\left(№\;Se+\;№\;De\right)\times \;\text{Wp}\;}\times \;10$$

where:

№ S*e* = number of surviving birds in *E. coli* inoculated group;

№ D*e* = number of dead birds in *E. coli* inoculated group;

№ Dp= number of dead birds in placebo inoculated group;

Wp = mean body weight of surviving birds in placebo inoculated group;

W*e* = mean body weight of surviving birds in *E. coli* inoculated group.

PLI can range from 0 (no difference in mean body weight and mortality between *E. coli* and placebo inoculated group) to 10 (100 % mortality in *E. coli* inoculated group and no mortality in the placebo group). This formula is only adequate in case the number of birds in the *E. coli* inoculated group and in the corresponding placebo group are approximately equal.

### Post mortem and lesion scoring

At the end of all the experiments, the surviving chickens were euthanized. Birds were first stunned by electrocution and killed by bleeding. Necropsy was performed on all the euthanized birds at the end of the experiments. The colibacillosis lesions were scored macroscopically following a previously established scoring system, first described by van Eck and Goren (1991). The mean lesions score (MLS) is assessed at the following anatomical sites: right and left thoracic air sac, pericardium and liver. Lesion scoring was performed using the following criteria to record the severity of the lesions: 0 = no lesions, 0.5 = one yellow / brown pinhead-sized spot indicative of inflammation, 1 = two or more pinhead-sized spots as just described, 2 = thin layer of fibrinous exudate on various locations, 3 = extensive thick layer of fibrinous exudate. The maximum score per bird is 12, calculated as the maximum score of 3 multiplied by the evaluation of four different anatomical sites. Postmortem examinations were also performed on the birds that died after the *E. coli* challenge to exclude causes of death other than colibacillosis.

### Statistical analysis

All statistical analysis was performed using GraphPad Prism version 10.4.1 (Prism, 2022). The statistical significance of differences in mortality rates and time of death were tested using the Cox proportional hazards regression model. In Experiment 1, mortality of each pure chicken line was compared to the commercial broiler line which functioned as a reference group and mortality was also compared between the other pure lines. In the second and third experiment the low dose inoculated group and *E. coli* inoculated groups served as reference group per chicken line, respectively. For each hazard ratio (HR) a 95 % confidence interval is given and HRs were considered significant if the entire 95 % confidence interval was either above or below 1.

The mean body weights at different ages during the experiment for the *E. coli*-inoculated groups and their placebo-inoculated counterparts were statistically compared. A mixed-effects model with the Geisser–Greenhouse correction was used, followed by Tukey’s multiple comparisons test, with individual variances computed for each comparison. The mean lesion scores were statistically compared using the Mann–Whitney test in Experiment 1, or the Kruskal–Wallis test followed by Dunn’s multiple comparison test in Experiments 2 and 3. Effects were considered to be significantly different at the 0.05 level (*P* < 0.05).

## Results

### Mortality

**Fig. 2:**
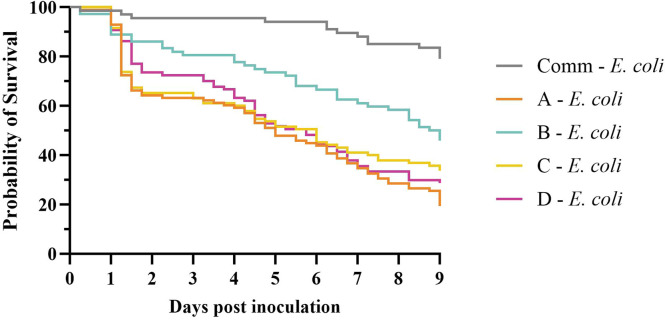
Survival curves from experiment 1. The probability of survival of the pure lines A, B, C, D and the commercial line following *E. coli* inoculation (10^7.1^ colony forming units/ bird) on day 8. Mock inoculated groups are not shown, as no or minimal mortality (≤ 3 birds/group) occurred in these groups.

*Experiment 1.* High Mortality rates, ranging from 54.2 to 80.6 %, were observed in birds from the pure lines following *E. coli* inoculation, whereas a lower mortality rate of 20.9 % was observed in birds from the commercial line (Table 1). This resulted in hazard ratios that were significantly higher in the pure lines compared to the commercial broiler line. The hazard ratios were 7.06 (95 % confidence interval (CI): 4.12 – 13.04), 3.38 (95 % CI: 1.88 – 6.45), 5.30 (95 % CI: 3.06 – 9.87) and 5.64 (95 % CI: 3.25 – 10.51) for pure lines A, B, C and D, respectively (Fig. 2). Hazard ratios did not statistically differ between pure lines A, C and D. However, hazard ratios did statistically differ for the pure lines A, C and D when compared to pure line B. The hazard ratios were respectively, 2.05 (95 % CI: 1.41 – 3.04), 1.57 (95 % CI: 1.05 – 2.35) and 1.67 (95 % CI: 1.12 – 2.51) for pure lines A, C and D. When the survival of males and females within each line was compared, no significant differences were observed (Supplementary Fig. 1).

**Table 1:** Summary of three experiments on the susceptibility to colibacillosis in different pure lines of broiler breeders and their four-way cross (commercial broilers). Mortality rates, body weights and mean lesions scores of chickens euthanized at the end of the experiment, and the production loss index (PLI) are given.

			Challenge					Surviving chickens			
Experiment	Chicken line	*n* = 1	IBV^2^	*E. coli*^3^		Mortality (%)		Bodyweight (±SD)	Mean lesion score		PLI
**1**	Commercial	66	−4	-		1.5		695 ± 73	0.1		na^5^
		67	-	High		20.9		624 ± 107	2.9		2.76
	A	90	-	-		1.1		501 ± 59	0.1		na
		98	-	High		80.6		384 ± 113	8.1		8.44
	B	69	-	-		4.3		545 ± 69	0.1		na
		72	-	High		54.2		305 ± 87	9.9		7.20
	C	94	-	-		0.0		565 ± 68	0.1		na
		95	-	High		66.3		541 ± 121	4.7		6.77
	D	84	-	-		1.2		531 ± 57	0.1		na
		87	-	High		71.3		387 ± 110	9.1		7.82
**2**	Commercial	74	-	-		0.0		559 ± 50	0.4		na
		71	-	Low		2.8		538 ± 54	0.5		0.65
		66	-	Medium		6.1		550 ± 101	2.8		0.76
		70	-	High		32.9		459 ± 170	6.6		4.49
	A	66	-	-		0.0		542 ± 58	0.3		na
		62	-	Low		19.4		521 ± 77	2.5		2.25
		68	-	Medium		33.8		491 ± 111	4.1		4.01
		70	-	High		90.0		387 ± 116	10.3		9.29
	B	64	-	-		0.0		504 ± 58	0.4		na
		68	-	Low		10.3		469 ± 93	2.9		1.65
		66	-	Medium		25.8		428 ± 113	5.4		3.70
		64	-	High		46.9		371 ± 150	7.5		6.09
**3**	Commercial	20	-	-		0.0		1273 ± 62	0.1		na
		20	-	High		15.0		1177 ± 188	3.0		2.14
		20	Yes	High		15.0		986 ± 163	7.9		3.42
	A	20	-	-		0.0		1283 ± 118	0.3		na
		19	-	High		52.6		1094 ± 257	3.9		6.16
		20	Yes	High		60.0		795 ± 205	8.4		7.52

1 Number of *E. coli*-inoculated birds.

2 Infectious bronchitis virus, vaccine strain H52, was inoculated intratracheally and oculonasally on day 16 at a dose of 10^5.5^ EID50 per bird.

3 *E. coli* was inoculated intratracheally on day 8 (experiments 1 and 2) or day 21 (experiment 3). High-dose inoculations were 10^7.2^, 10^7.1^, and 10^7.0^ colony forming units (CFU) per bird for experiment 1, 2, and 3, respectively. Medium (10^6.1^ CFU per bird) and low (10^5.1^ CFU per bird) doses were used in experiment 2.

4 Phosphate-buffered saline inoculations were performed at the same time points as IBV or *E. coli* inoculations.

5 Not applicable; PLI is calculated based on the control group (see ‘Materials and Methods’).

Mortality was observed as early as 6 hours post-inoculation in birds from the commercial line and pure lines A and B. Acute mortality was observed within 1.5 days post-inoculation (dpi). In the subsequent period - from 1.5 dpi up to 3.5-4 dpi in the pure lines and 5 dpi in the commercial line - only a few birds died. After this phase, mortality gradually increased in all *E. coli*-inoculated groups (Fig. 2). In all control groups 6 out of 403 birds died after PBS inoculation (Supplementary Table 1), however, no macroscopic lesions related to colibacillosis were observed in these birds.

*Experiment 2*. Similar mortality rates were observed in the high inoculation dose groups when compared with the same chicken lines in Experiment 1 (Table 1). In the second experiment, the hazard ratio for pure line B compared with the commercial line was 1.70 (95 % CI: 0.84–3.56), which was not statistically significant. In contrast, the risk of mortality for chickens from pure line A was significantly higher, with a hazard ratio of 6.72 (95 % CI: 3.55–13.48), compared with the commercial line.

**Fig. 3:**
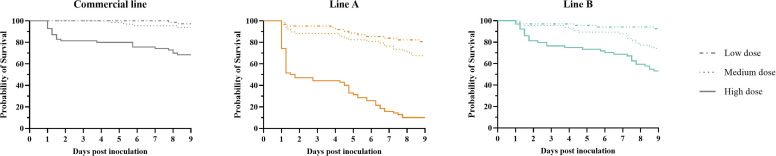
Survival curves from experiment 2. The probability of survival of the pure lines A, B, and the commercial line following *E. coli* inoculation with three different dosages, 10^5.1^ (low), 10^6.1^ (medium) and 10^7.1^ (high) colony forming units per bird. Mock inoculated groups are not shown, as no mortality occurred in these groups.

In the groups inoculated with a high inoculation dose, a phase of acute mortality (up to 1.5-2.0 dpi), followed by a second period of mortality (4.25-6.0 dpi onwards) was also observed in this experiment. However, the acute mortality phase was not clearly observed in the in the groups that received medium or low inoculation doses (Fig. 3). In all lines, the hazard ratios for the high inoculation dose groups were significantly higher when compared to the low inoculation dose groups within the same chicken line. The hazard ratios were 13.55 (95 % CI: 3.99 – 84.59), 10.85 (95 % CI: 6.00 – 21.36) and 8.28 (95 % CI: 3.50 – 24.31) for the commercial line and pure lines A and B, respectively. For the medium inoculation dose groups, hazard ratios in the commercial line and line A were not significantly higher than in the low inoculation dose groups. Only in pure line B was the hazard ratio of 3.79 (95 % CI: 1.50 – 11.52) significantly higher than that of the low inoculation dose group. All birds survived in the PBS-inoculated groups (Supplementary Table 2).

**Fig. 4:**
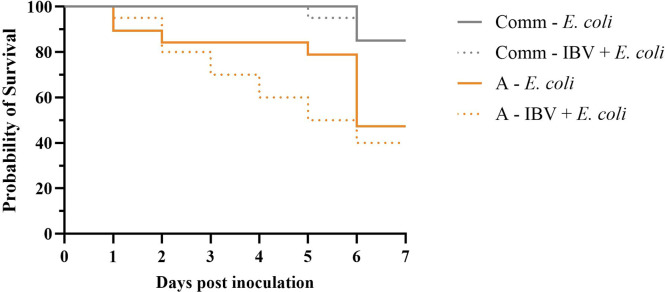
Survival curves from experiment 3. The probability of survival of the pure line A and the commercial line following dual infection with infectious bronchitis virus (IBV) H52, administrated five days prior to *E. coli* inoculation (10^7.0^ colony forming units / bird). Mock inoculated groups are not shown, as no mortality occurred in these groups.

*Experiment 3*. Mortality rates between the groups inoculated with *E. coli* alone or with IBV and *E. coli* did not differ. In pure line A mortality rates were 52.6 and 60.0 %, respectively, and in both groups of the commercial broiler line the mortality rate was 15.0 % (Table 1). The hazard ratios of the IBV and *E. coli* inoculated groups did not significantly differ from the *E. coli* inoculated groups (Fig. 4).

### Body weights

**Fig. 5:**
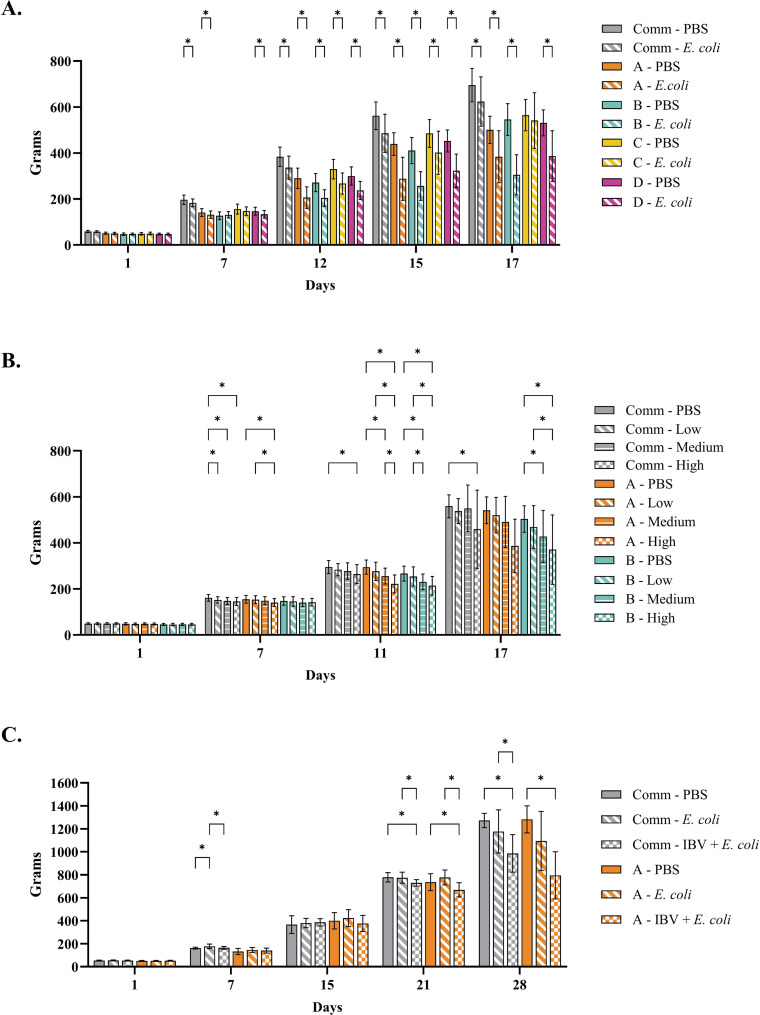
The bodyweights of birds at various timepoints during the experiments are presented. In Experiment 1 (Fig. 2.A), pure lines A, B, C, D and commercial broilers (Comm) were weighted on days 1, 7, 12, 15 and 17. All birds were intratracheally inoculated on day 8 with either *E. coli* or physiological buffered saline (PBS). Pure lines A, B, and the commercial line were used in Experiment 2 (Fig. 2.B) and weighted at days 1, 7, 11 and 17. Birds were mock-inoculated (PBS) or *E. coli* inoculated with three different doses: high (H), medium (M) and low (L), corresponding to 10^7.1^, 10^6.1^, 10^5.1^ colony forming units per bird, respectively. In Experiment 3 (Fig. 2.C), pure-line A and commercial broilers were inoculated with infectious bronchitis virus (IBV) H52 and *E. coli*, with *E. coli* alone or mock-inoculated with PBS. Inoculations were performed at day 16 (IBV or PBS) day 21 (*E. coli* or PBS) and birds were weighted at day 1, 7, 15, 21, and 28. Asterisks indicate a *P* value less than 0.05, representing a statistically significant difference.

In Fig. 5, the mean bodyweights of the birds in all three experiments are presented. Before inoculations with *E. coli* (day 8) or IBV(day 16), significant differences in body weights between groups of the commercial line (all experiments), pure line A (Experiment 1 and 2) and pure line D (Experiment 1) were observed.

*Experiment 1*. The mean body weight of the *E. coli*-inoculated group was significantly lower for each chicken line after inoculation (day 12 onwards) (*P* < 0.05) compared to the control birds. Only the body weight of birds from pure line C, at the end of the experiment did not statistically differ from each other (*P* = 0.99) (Fig. 5A). No statistical differences in body weights were observed between the male and females (Supplementary Fig. 2).

*Experiment 2.* The *E. coli*-inoculation dose had an impact on the body weight of the birds. In general, the higher the *E. coli* inoculation dose, the lower the body weights (Fig. 5B). In the commercial line, no statistical differences in body weights were observed between the PBS and the low and medium *E. coli*-inoculated groups, only between the PBS-inoculated groups and the high dose inoculated group (*P* = 0.01).Significant differences were observed at day 11 for both pure lines tested (*P* < 0.05). However, at the end of the experiment (day 17), significant differences were only observed in pure line B between the groups inoculated with different doses and the PBS-inoculated group (Fig. 5B).

*Experiment 3*. After IBV inoculation and before the *E. coli* inoculation, significant differences in body weight were observed between the IBV-inoculated group and the other groups within the same chicken line (*P* < 0.05). Following *E. coli* inoculation, differences in body weights were more pronounced. In the groups receiving both IBV and *E. coli*, body weights at the end of the experiment were significantly higher in the commercial line compared to the pure line A and were both significantly lower (*P* ≤ 0.001) than those of the PBS-inoculated birds (Fig. 5 and Suppl. Table 3). Although the *E. coli*-inoculated birds had lower body weights than the PBS-inoculated birds, these were not statistically different. Only in the commercial line, the *E. coli*-inoculated birds had significant higher body weights when compared to birds inoculated with both IBV and *E. coli*.

### Production loss index

PLI is an indicator for the loss in body weight due to growth depression and mortality and the PLIs are given in Table 1. In all experiments, the PLI of the *E. coli*-inoculated birds of the commercial line were lower compared to groups of the pure lines with the same treatment. The highest PLI was observed in groups of pure line A. The pure line with the lowest PLI in Experiment 1 was line C, although mortality was comparable with pure lines A and D, the body weights of the surviving birds did not differ from the PBS inoculated birds.

### Mean lesion scoring

**Fig. 6:**
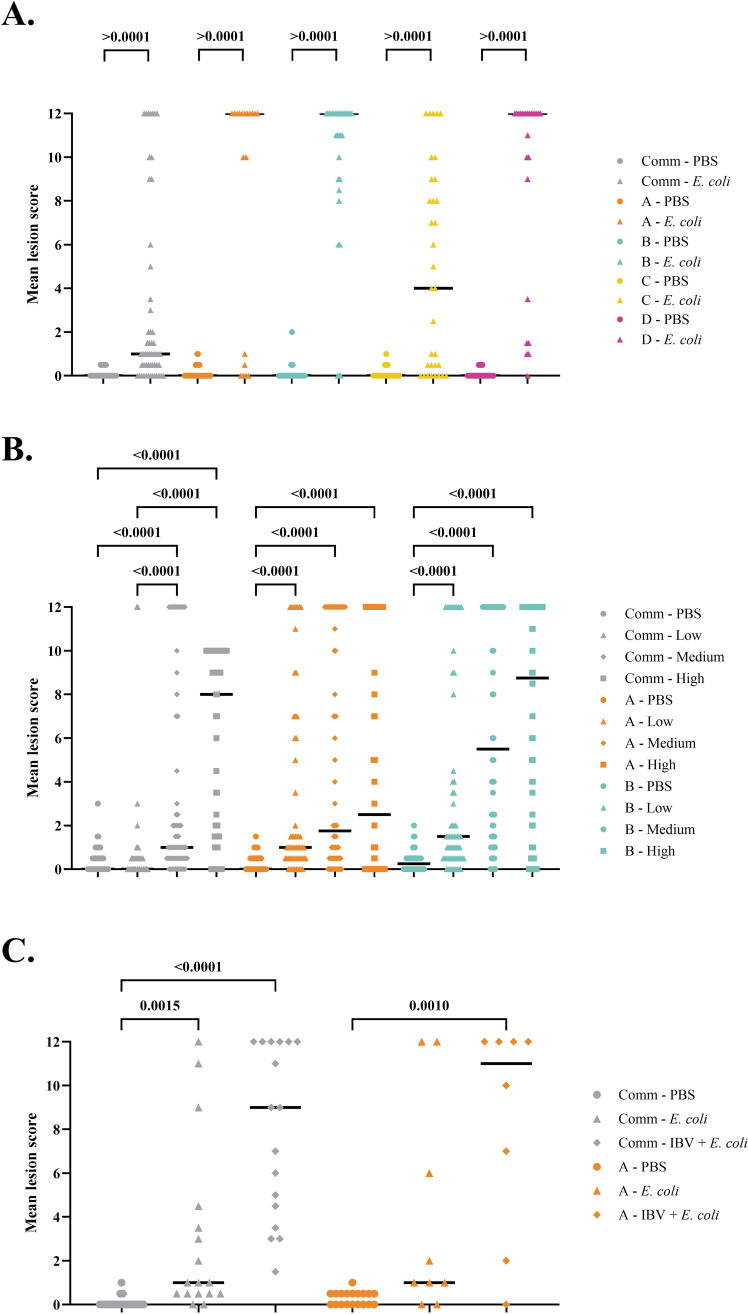
Mean lesion scores assessed in surviving birds at the end of each experiment. Each dot represents an individual bird and the median per group are indicated with a solid line. In Experiment 1 (Fig. 6.A), pure lines A, B, C, D and commercial broilers (Comm) were inoculated with either *E. coli* or physiological buffered saline (PBS). In Experiment 2 (Fig. 6.B), pure lines A, B, and the commercial line were used and were mock-inoculated (PBS) or *E. coli* inoculated with three different doses: high (H), medium (M) and low (L), corresponding to 10^7.1^, 10^6.1^, 10^5.1^ colony forming units per bird, respectively. In Experiment 3 (Fig. 6.C), pure-line A and commercial broilers were inoculated with infectious bronchitis virus (IBV) and *E. coli*, with *E. coli* alone or mock-inoculated with PBS. Inoculations were performed at day 16 (IBV or PBS) or day 21 (*E. coli* or PBS) and birds were weighted on day 1, 7, 15, 21, and 28.

Mean lesion scoring was only performed on all surviving chicks that were euthanized at the end of the experiments. In all chicken lines, the mean lesion score (MLS) was significantly higher in the *E. coli*-inoculated groups compared to the controls (*P* < 0.001). In Experiment 1, the highest MLS of 9.9 was observed in pure line B, followed pure lines D and A with scores of 9.1 and 8.1, respectively. In pure line C, the MLS was 4.7, also no significant differences in body weight was observed compared to the control group (Fig. 6A and Table 1)*.* The MLSs of males and females did not significantly differ across all chicken lines, except for pure line B, where female birds had significantly lower MLSs than male birds (Supplementary Fig. 3).

*Experiment 2*. In surviving chickens of pure lines A and B, MLS scores were significantly higher (*P* < 0.001) in the *E. coli*-inoculated groups compared to the PBS-inoculated groups. However, no significant difference were observed among the three inoculation doses used. Commercial broilers inoculated with the low dose of *E. coli* and the controls did not significantly differ from each other, but did significantly differ with the groups inoculated with an medium or high dose (Fig. 6B).

IBV combined with *E. coli* inoculations resulted in significant higher MLSs compared to PBS-inoculated birds (*P* < 0.001). *E. coli* inoculation alone also led to increased MLSs, however, the difference was statistically significant compared to the control group only in the commercial line (Fig. 6C).

## Discussion

Susceptibility to colibacillosis may differ between genetic lines, and selecting for resistance to colibacillosis through genetic selection may reduce the disease's impact in commercial broilers. Therefore, in this study the susceptibility of four pure broiler breeder lines and a four-way cross of these lines – representing a commercial broiler line – to colibacillosis was assessed. This study revealed distinct differences in susceptibility among the pure lines. These differences were not dose-, or sex-dependent and remained consistent when a dual IBV–*E. coli* infection model was applied.

In this study, susceptibility to colibacillosis was evaluated based on mortality, mean body weights, and lesion scores observed during post-mortem examination. Based on all aforementioned parameters, the commercial broiler line was the least susceptible to colibacillosis. These striking differences when compared to the tested pure lines, who are the genetic ancestors of these commercial broiler, can most likely be attributed to the heterosis effect. Heterosis is the improvement of traits in offspring gained by crossing two unrelated populations (Goodwin, et al., 1956; Williams, et al., 2002). Our results are in disagreement with the results of Ask, et al. (2006) were they found an absence of (positive), and even negative, heterosis in a two-way cross of the tested pure lines. However, the effect of heterosis is greater in four-way crosses than in two-way crosses due to an further increase in heterozygosity (Cavero, et al., 2009; Dickerson, 1964).

Susceptibility to disease is often not defined by a single parameter, but rather by a combination of clinical indicators influenced by the disease. This complexity makes it challenging to define the disease susceptibility of different genotypes, such as in the case of colibacillosis. In the present study, when susceptibility was solely based on mortality rates, pure line A appeared to be the most susceptible to avian pathogenic *E. coli*, closely followed by lines C and D (Table 1 and Fig. 2). In contrast, pure line B exhibited the lowest mortality rates. However, by the end of the experiment, the body weights of inoculated birds were significantly lower compared to their control counterparts. Meanwhile, surviving chickens from pure line C that were inoculated with *E. coli* did not show statistically significant differences in body weight compared to PBS-inoculated controls. Furthermore, *E. coli*-inoculated birds exhibited significantly higher MLSs compared to the controls. These results suggest that line C might be of particular interest for breeding programs, as surviving birds develop colibacillosis (as indicated by high MLSs), but maintain normal body weight.

Combining clinical indicators into a single parameter, can be used to determine the susceptibility to colibacillosis among different genetic lines. Both growth retardation and mortality are of great economic importance in broiler production and were combined into a Production Losses Index (PLI) by van Eck and Goren (1991). In cases where mortality differences are relatively small, the PLI may serve as a useful parameter for assessing susceptibility to colibacillosis. Although,the mortality rate in pure line B was significantly lower compared to, for example, pure line C, when PLIs were compared between the two strains, the lowest PLI among the pure lines was observed in line C. This index may also be relevant for assessing the impact of diseases on broiler production in cases where both growth and mortality are affected. It is important to note that not all economically relevant clinical parameters are included in the calculation of the PLI. Further improvements could be made by adjusting the index to account for other important production losses, such as carcass rejections at the slaughterhouse due to colisepticemic lesions or potential increases in feed conversion rates.

Selection traits are mostly based on economic importance, colibacillosis is not the sole disease in poultry industry. Defining the major disease challenges in poultry and incorporating this as selection traits in breeding programs could help improve disease resistance in broilers. However, the economic importance of avian diseases extends beyond colibacillosis and includes viral, bacterial and parasitic challenges. Resistance to viral infections depends on different immune pathways compared to those involved in bacterial resistance. The latter is often mediated by innate immune mechanisms such as phagocytosis, Toll-like/C-type lectin receptor pathways, and complement activation (Alber, et al., 2021), whereas antiviral immunity relies more heavily on adaptive responses, including interferon signaling, natural killer cells, and cytotoxic T lymphocytes (Mueller and Rouse, 2008). From a heritability perspective, these pathways show limited overlap, although certain genes—such as those involved in the MHC or cytokine signaling—contribute to both bacterial and viral immune responses (Flajnik and Kasahara, 2010; Silva and Gallardo, 2020).

In general, higher inoculation dose led to more severe effects on mortality, body weights and MLSs, as was observed in Experiment 2. This dose-dependent effect is consistent with findings from other experimental *E. coli* infections (Alber, et al., 2019; Landman, et al., 2021). However the inoculation doses used were relatively high (10^5.1^–10^7.2^ cfu per bird). It remains difficult to determine whether these *E. coli* doses accurately reflect field conditions. In poultry houses, *E. coli* concentrations of 10^2^–10⁴ bacteria per m³ air have been reported, leading to an estimated uptake of 10¹–10³ cfu per hour per bird (Chinivasagam, et al., 2009; Fedde, et al., 1998). The estimated uptake of *E. coli* in the field is continuous rather than instantaneously, and lower than the inoculation doses used in the present study (10^5.1^–10^7.2^ cfu per bird). Despite this, the study successfully reproduced a disease pattern similar to natural colibacillosis in commercial broilers, with mild mortality, growth retardation, and a high incidence of airsacculitis.

In this study, chicks were assigned to experimental groups with equal numbers of males and females on day 1 and kept under identical conditions in the same experimental unit, resulting in similar mean body weights before inoculation. However, significant differences (*P* < 0.05) between groups of the commercial line (all experiments), pure line A (Experiment 1 and 2) and pure line D (Experiment 1) were observed before inoculation (Fig. 5). Beside biological variation, the authors do not have a logical explanation for these discrepancies. Future research should include sufficient replicates to exclude any group effects. Although the differences in body weights at the start of the experiment were statistically significant, their impact on body weights after *E. coli* inoculation is considered negligible. In previous studies, the effect of *E. coli* inoculation on body weight has been shown to be severe, and these effects are expected to be far greater than the initial differences observed before inoculation.

The *E. coli*-infection model used in this study was introduced in 1991 (van Eck and Goren, 1991). In the first experimental trials (Matthijs, et al., 2003), *E. coli* was re-isolated from half of the collected samples. In all positive cases, the re-isolated *E. coli* was identical to the strain used for inoculation. Dwars, et al. (2009) demonstrated, through immunohistochemical staining, that the inoculated *E. coli* was abundantly present in all examined organs, particularly within the purulent exudate. Re-isolation by culturing is only possible if *E. coli* has not yet been eliminated by the immune system, which explains why re-isolation was successful in only half of the cases. For the reasons mentioned above, no re-isolation was performed in the later series of animal experiments that used the same experimental model with the same *E. coli* strain (Berghof, et al., 2019a; Wijnen, et al., 2022). Nevertheless, the observed diseased birds, mortality rate, and postmortem findings confirmed that the *E. coli* inoculation was successful.

In this infection model, mortality is the most severe outcome. Determining the MLS in animals that died is not meaningful, as deaths occurring within 48 hours post-inoculation are typically due to septicemia (Matthijs, et al., 2003), and the animals die too quickly to develop macroscopical visible exudate. From previous *E. coli* animal experiments, it is clear that the mean lesion score (MLS) is correlated with body weight. The highest *E. coli* doses resulted in the lowest body weight uniformity and the highest mean lesion scores (Matthijs, et al., 2003). The increase in mean lesion scores was mainly due to a higher number of birds with systemic colibacillosis (*i.e.*, heart and liver affected), rather than an increase in the percentage of affected birds

The commercial broilers, a four-way cross of the tested pure lines, demonstrated superior resistance to colibacillosis compared to the pure lines in all experiments. Among the pure lines, genetic differences in susceptibility to colibacillosis were observed. These differences indicate that genetic selection for resistance to colibacillosis might be feasible. Such selection could contribute to a reduced incidence of colibacillosis in the field, leading to lower costs, improved animal welfare and health, and a decreased reliance on antibiotics.

## CRediT authorship contribution statement

**T.T.M. Manders:** Writing – review & editing, Writing – original draft, Visualization, Validation, Supervision, Methodology, Investigation, Formal analysis, Data curation, Conceptualization. **A. Papanikolaou:** Writing – review & editing, Writing – original draft, Visualization, Validation, Formal analysis, Data curation, Conceptualization. **J.J. de Louwere:** Writing – review & editing, Writing – original draft, Visualization, Validation, Methodology, Conceptualization. **M.G.R. Matthijs:** Writing – review & editing, Writing – original draft, Supervision, Resources, Project administration, Methodology, Investigation, Funding acquisition, Formal analysis, Data curation, Conceptualization.

## Disclosures

The authors declare that they have no known competing financial interests or personal relationships that could have appeared to influence the work reported in this paper.

## Appendix Supplementary materials

Image, application 1

Image, application 2

Image, application 3

Image, application 4

Image, application 5

Image, application 6

Image, application 7
