# Supplementary material for: Quantitative assessment and comparison of susceptibility to colibacillosis in pure lines of broiler breeders and their commercial offspring
Source: Poult Sci. 2025 Aug 24;104(11):105722. doi: 10.1016/j.psj.2025.105722 (PMC12451321; doi:10.1016/j.psj.2025.105722)
Supplement: Supplementary file 1 [file mmc1.docx]

**Supplementary Figure 1**. The bodyweights of male and females used in experiment 1 are presented for days 1, 7, 12, 15 and 17. Birds of pure lines A, B, C, D and commercial broilers (Comm) were inoculated on day 8 with either *E. coli* or physiological buffered saline (PBS). An asterisk (*) indicates a statistically significant difference (P < 0.05).

**Supplementary Figure 2.** Survival curves from Experiment 1, depicting male and females of pure line A, B, C, D and commercial broilers following *E. coli* inoculation on day 8. Mock inoculated groups are not shown, as no or minimal mortality occurred in these groups.

**Supplementary Figure 3.**  Mean lesion scores assessed in surviving females and males of pure line A, B, C, D and commercial broilers at the end of Experiment 1. Each dot represents an individual bird and the median per group is indicated with a solid line.
