# Supplementary figures and images for: Quantitative assessment and comparison of susceptibility to colibacillosis in pure lines of broiler breeders and their commercial offspring
Source: Poult Sci. 2025 Aug 24;104(11):105722. doi: 10.1016/j.psj.2025.105722 (PMC12451321; doi:10.1016/j.psj.2025.105722)

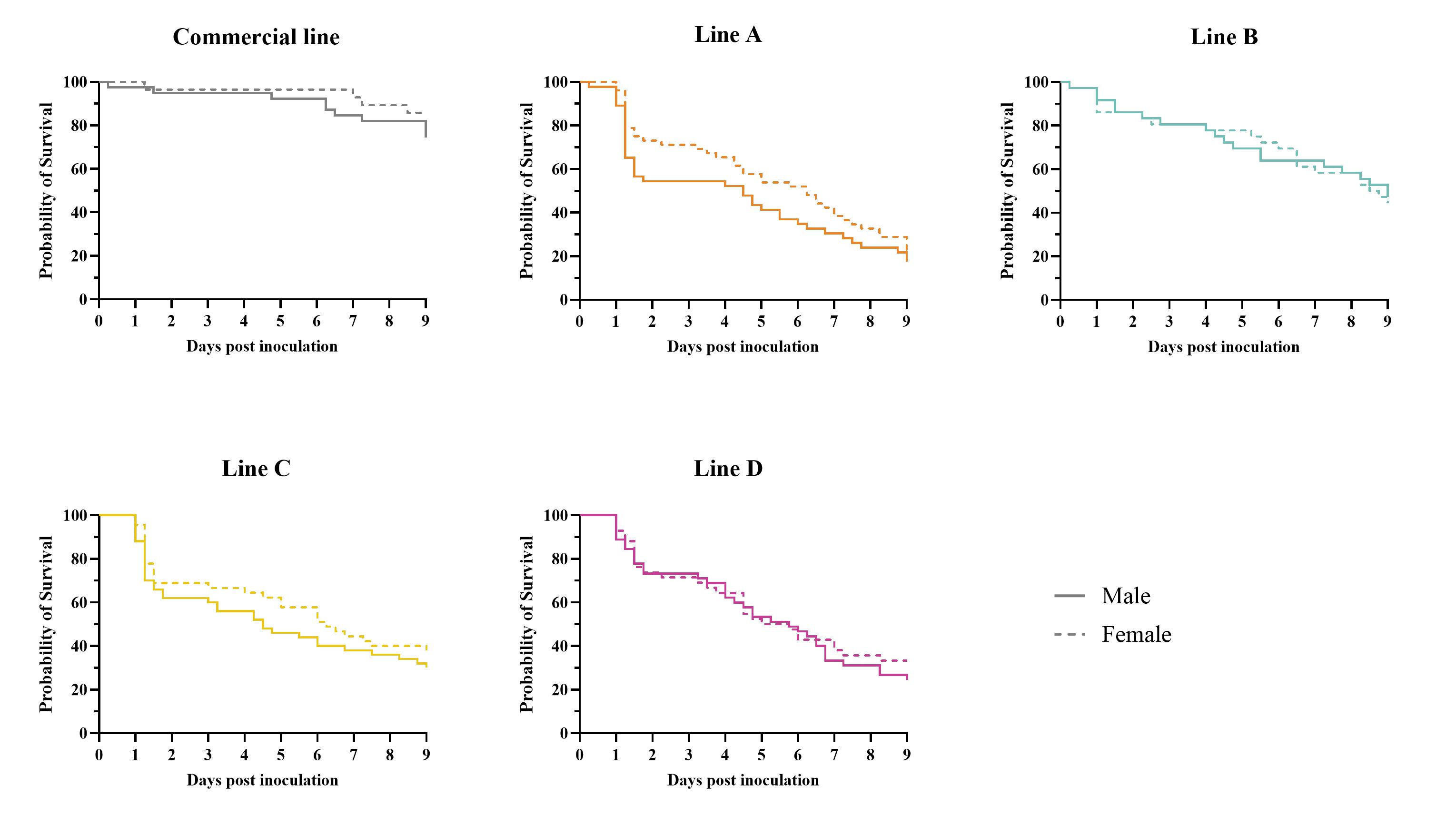

Supplement: Supplementary file 2 [file mmc2.zip › mmc2.jpg]

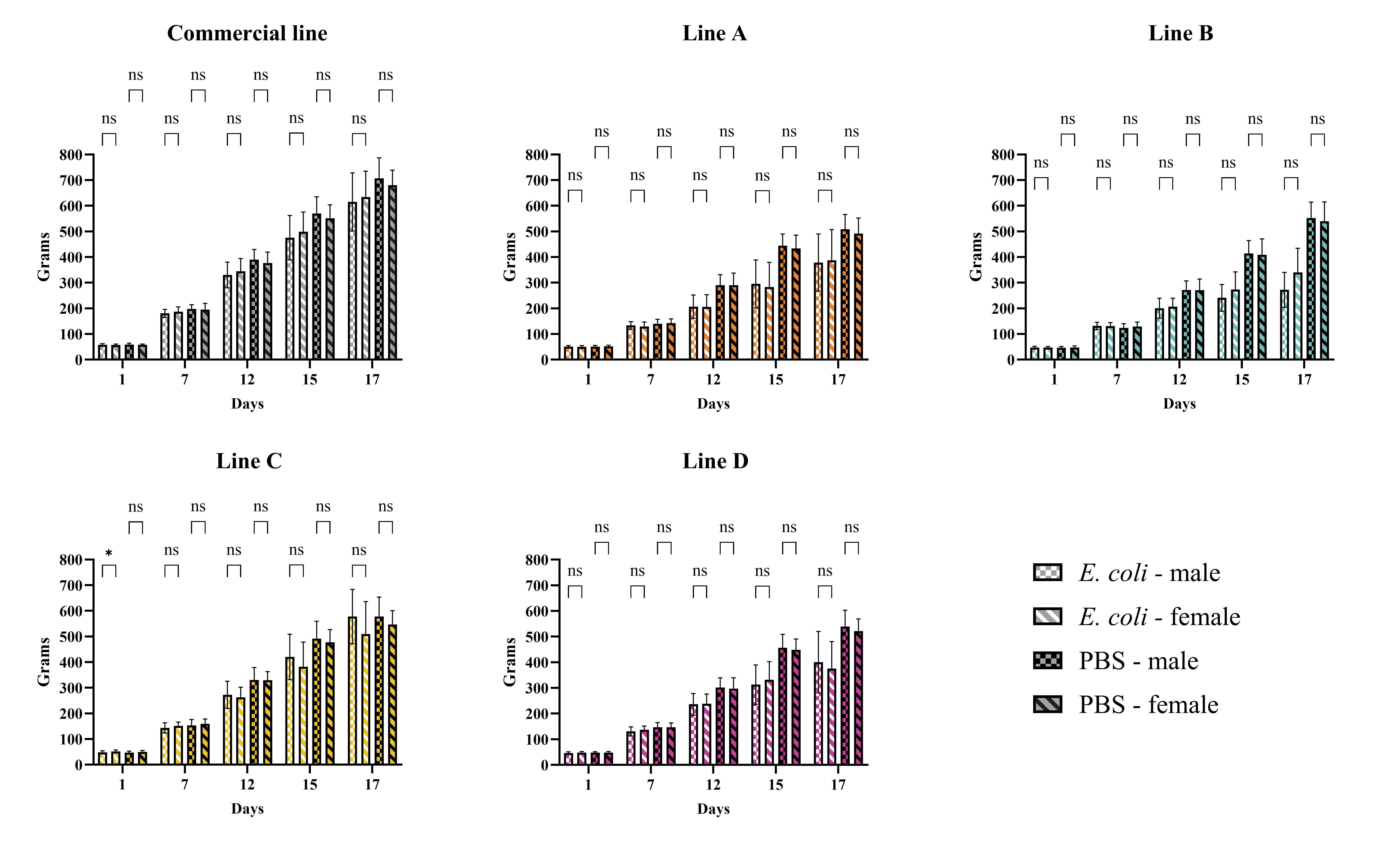

Supplement: Supplementary file 3 [file mmc3.zip › mmc3.jpg]

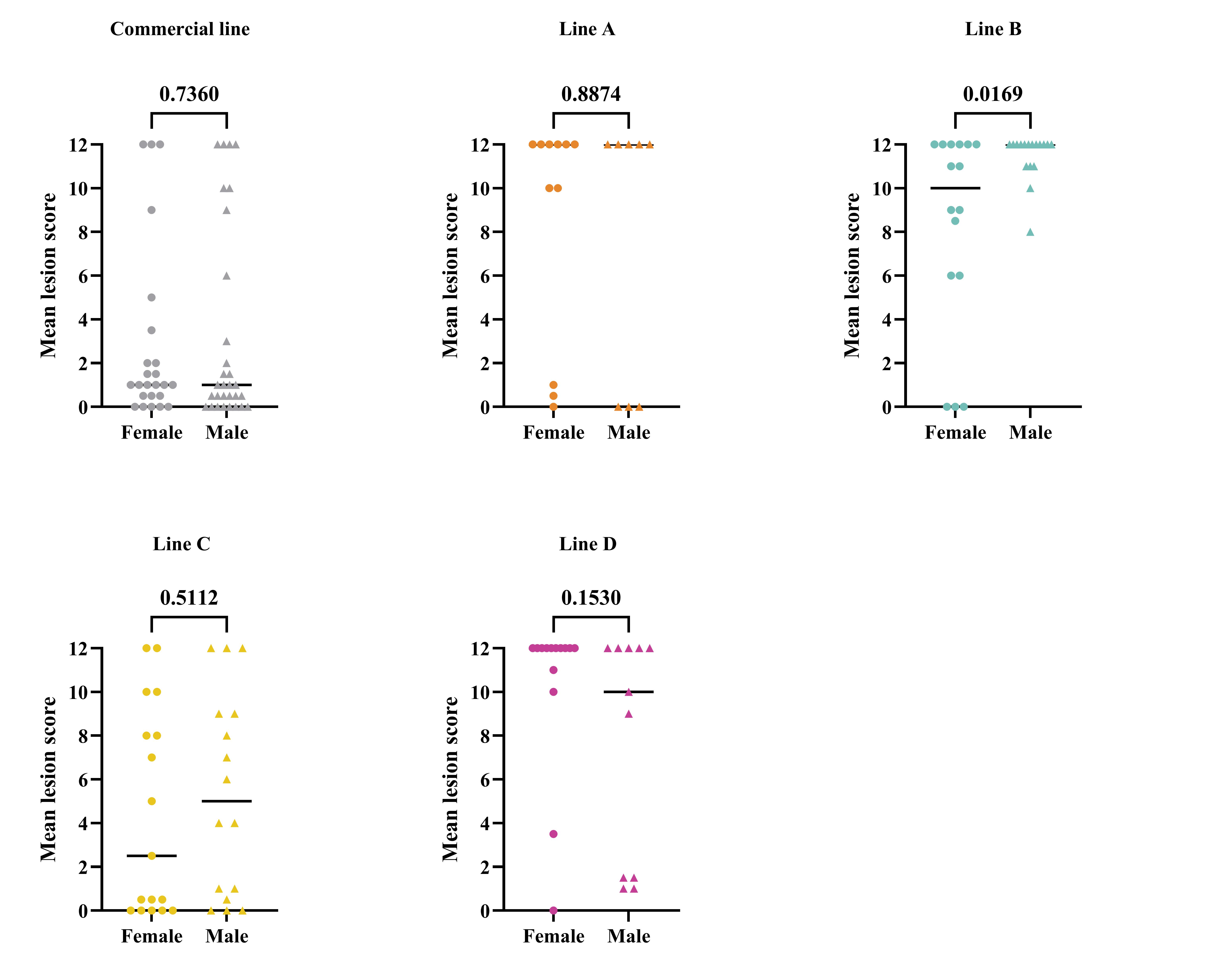

Supplement: Supplementary file 4 [file mmc4.zip › mmc4.jpg]
