# Supplementary material for: Quantitative assessment and comparison of susceptibility to colibacillosis in pure lines of broiler breeders and their commercial offspring
Source: Poult Sci. 2025 Aug 24;104(11):105722. doi: 10.1016/j.psj.2025.105722 (PMC12451321; doi:10.1016/j.psj.2025.105722)
Supplement: Supplementary file 5 [file mmc5.docx]

**Supplementary Table 1**. Overview of the results of experiment 1.

|  |  |  |  |  |  | Day | | | | | | | | | | | | | |  | Surviving chickens at end of experiment | |
| --- | --- | --- | --- | --- | --- | --- | --- | --- | --- | --- | --- | --- | --- | --- | --- | --- | --- | --- | --- | --- | --- | --- |
|  |  |  |  |  |  | 1 | |  | 7 | |  | 12 | |  | 15 | |  | 17 | |  |  |  |
| Chicken line | *E. coli* inoculation | Gender | Number of birds allocated to group | Number of Inoculated birds |  | N=^1^ | BW ±SD^2^ |  | N= | BW ± SD |  | N= | BW ± SD |  | N= | BW ± SD |  | N= | BW ± SD |  | N= | Mean lesion score |
| **Commercial** | **+** | ♂ | 40 | 39 |  | 40 | 59 ± 4 |  | 39 | 181 ± 15 |  | 37 | 330 ± 50 |  | 32 | 476 ± 86 |  | 29 | 615 ± 113 |  | 29 | 3.0 |
|  |  | ♀ | 28 | 28 |  | 28 | 57 ± 4 |  | 28 | 187 ± 18 |  | 27 | 344 ± 50 |  | 25 | 499 ± 77 |  | 24 | 634 ± 101 |  | 24 | 2.8 |
|  |  | **♂ + ♀** | **68** | **67** |  | **68** | **58 ± 4** |  | **67** | **183 ± 17** |  | **64** | **336 ± 50** |  | **57** | **486 ± 83** |  | **53** | **624 ± 107** |  | **53** | 2.9 |
|  | **-** | ♂ | 38 | 38 |  | 38 | 59 ± 5 |  | 38 | 198 ± 17 |  | 37 | 390 ± 40 |  | 37 | 571 ± 64 |  | 37 | 707 ± 80 |  | 37 | 0.1 |
|  |  | ♀ | 28 | 28 |  | 28 | 57 ± 4 |  | 28 | 195 ± 24 |  | 28 | 376 ± 44 |  | 28 | 551 ± 53 |  | 28 | 680 ± 59 |  | 28 | 0.1 |
|  |  | **♂ + ♀** | **66** | **66** |  | **66** | **58 ± 5** |  | **66** | **197 ± 20** |  | **65** | **384 ± 42** |  | **65** | **562 ± 60** |  | **65** | **695 ± 73** |  | **65** | 0.1 |
| **A** | **+** | ♂ | 47 | 46 |  | 47 | 51 ± 4 |  | 46 | 134 ± 15 |  | 24 | 207 ± 45 |  | 13 | 295 ± 93 |  | 8 | 379 ± 111 |  | 8 | 7.5 |
|  |  | ♀ | 52 | 52 |  | 52 | 50 ± 5 |  | 52 | 130 ± 17 |  | 31 | 206 ± 49 |  | 20 | 284 ± 96 |  | 11 | 387 ± 120 |  | 11 | 8.5 |
|  |  | **♂ + ♀** | **99** | **98** |  | **99** | **50 ± 5** |  | **98** | **132 ± 16** |  | **55** | **206 ± 47** |  | **33** | **288 ± 94** |  | **19** | **384 ± 113** |  | **19** | 8.1 |
|  | **-** | ♂ | 48 | 47 |  | 48 | 51 ± 5 |  | 47 | 140 ± 18 |  | 47 | 290 ± 42 |  | 47 | 444 ± 46 |  | 47 | 509 ± 58 |  | 47 | 0.1 |
|  |  | ♀ | 45 | 43 |  | 45 | 52 ± 5 |  | 43 | 143 ± 16 |  | 42 | 290 ± 48 |  | 42 | 434 ± 52 |  | 42 | 492 ± 61 |  | 42 | 0.1 |
|  |  | **♂ + ♀** | **93** | **90** |  | **93** | **51 ± 5** |  | **90** | **141 ± 17** |  | **89** | **290 ± 44** |  | **89** | **439 ± 49** |  | **89** | **501 ± 59** |  | **89** | 0.1 |
| **B** | **+** | ♂ | 38 | 36 |  | 38 | 47 ± 5 |  | 36 | 132 ± 14 |  | 27 | 201 ± 39 |  | 22 | 241 ± 52 |  | 17 | 272 ± 68 |  | 17 | 11.5 |
|  |  | ♀ | 36 | 36 |  | 36 | 48 ± 4 |  | 36 | 131 ± 13 |  | 29 | 207 ± 33 |  | 21 | 273 ± 69 |  | 16 | 341 ± 94 |  | 16 | 8.3 |
|  |  | **♂ + ♀** | **74** | **72** |  | **74** | **47 ± 4** |  | **72** | **131 ± 14** |  | **56** | **204 ± 36** |  | **43** | **257 ± 62** |  | **33** | **305 ± 87** |  | **33** | 9.9 |
|  | **-** | ♂ | 38 | 31 |  | 38 | 47 ± 5 |  | 31 | 124 ± 17 |  | 30 | 271 ± 36 |  | 29 | 413 ± 51 |  | 29 | 552 ± 62 |  | 29 | 0.1 |
|  |  | ♀ | 39 | 38 |  | 39 | 48 ± 6 |  | 38 | 129 ± 17 |  | 37 | 271 ± 43 |  | 36 | 409 ± 62 |  | 37 | 510 ± 75 |  | 37 | 0.1 |
|  |  | **♂ + ♀** | **77** | **69** |  | **77** | **47 ± 5** |  | **69** | **127 ± 17** |  | **67** | **271 ± 39** |  | **65** | **411 ± 57** |  | **66** | **545 ± 69** |  | **66** | 0.1 |
| **C** | **+** | ♂ | 52 | 50 |  | 52 | 49 ± 6 |  | 50 | 144 ± 20 |  | 26 | 273 ± 53 |  | 19 | 420 ± 89 |  | 15 | 578 ± 106 |  | 15 | 4.9 |
|  |  | ♀ | 45 | 45 |  | 45 | 52 ± 6 |  | 45 | 151 ± 15 |  | 29 | 263 ± 39 |  | 19 | 382 ± 97 |  | 17 | 509 ± 127 |  | 17 | 4.5 |
|  |  | **♂ + ♀** | **97** | **95** |  | **97** | **50 ± 6** |  | **95** | **147 ± 18** |  | **55** | **268 ± 46** |  | **38** | **401 ± 94** |  | **32** | **541 ± 121** |  | **32** | 4.7 |
|  | **-** | ♂ | 53 | 52 |  | 53 | 47 ± 6 |  | 52 | 153 ± 23 |  | 52 | 330 ± 49 |  | 52 | 492 ± 68 |  | 52 | 579 ± 75 |  | 52 | 0.1 |
|  |  | ♀ | 44 | 42 |  | 44 | 50 ± 5 |  | 42 | 160 ± 19 |  | 42 | 330 ± 36 |  | 42 | 477 ± 50 |  | 42 | 547 ± 53 |  | 42 | 0.1 |
|  |  | **♂ + ♀** | **97** | **94** |  | **97** | **49 ± 6** |  | **94** | **156 ± 21** |  | **94** | **330 ± 42** |  | **94** | **485 ± 61** |  | **94** | **565 ± 68** |  | **94** | 0.1 |
| **D** | **+** | ♂ | 46 | 45 |  | 46 | 47 ± 4 |  | 45 | 131 ± 18 |  | 29 | 236 ± 42 |  | 15 | 313 ± 77 |  | 11 | 400 ± 120 |  | 11 | 7.6 |
|  |  | ♀ | 42 | 42 |  | 42 | 48 ± 4 |  | 42 | 137 ± 15 |  | 26 | 239 ± 37 |  | 15 | 332 ± 70 |  | 14 | 376 ± 105 |  | 14 | 10.3 |
|  |  | **♂ + ♀** | **88** | **87** |  | **88** | **47 ± 4** |  | **87** | **134 ± 17** |  | **55** | **237 ± 40** |  | **30** | **322 ± 73** |  | **25** | **387 ± 110** |  | **25** | 9.1 |
|  | **-** | ♂ | 46 | 43 |  | 46 | 48 ± 4 |  | 43 | 147 ± 18 |  | 43 | 302 ± 38 |  | 43 | 456 ± 53 |  | 43 | 539 ± 64 |  | 43 | 0.1 |
|  |  | ♀ | 42 | 41 |  | 42 | 47 ± 5 |  | 41 | 147 ± 17 |  | 40 | 298 ± 42 |  | 40 | 448 ± 43 |  | 40 | 522 ± 47 |  | 40 | 0.1 |
|  |  | **♂ + ♀** | **88** | **84** |  | **88** | **48 ± 4** |  | **84** | **147 ± 17** |  | **83** | **300 ± 40** |  | **83** | **453 ± 48** |  | **83** | **531 ± 57** |  | **83** | 0.1 |

^1^Number of birds

^2^Body weight ± standard deviation
