# Supplementary material for: Quantitative assessment and comparison of susceptibility to colibacillosis in pure lines of broiler breeders and their commercial offspring
Source: Poult Sci. 2025 Aug 24;104(11):105722. doi: 10.1016/j.psj.2025.105722 (PMC12451321; doi:10.1016/j.psj.2025.105722)
Supplement: Supplementary file 6 [file mmc6.docx]

**Supplementary Table 2.** Overview of the results of experiment 2.

|  |  |  |  |  | Bodyweight at day (grams) | | | | | | | | | | |  | Surving chickens at end of experiment | |
| --- | --- | --- | --- | --- | --- | --- | --- | --- | --- | --- | --- | --- | --- | --- | --- | --- | --- | --- |
|  |  |  |  |  | 1 | |  | 7 | |  | 11 | |  | 17 | |  |  |  |
| Chicken line | *E. coli* dose | Number of birds allocated to group | Number of Inoculated birds |  | N=^2^ | BW ± SD^3^ |  | N= | BW ± SD |  | N= | BW ± SD |  | N= | BW ± SD |  | N= | Mean lesions score |
| **Commercial** | **-** | 77 | 74 |  | 77 | 49 ± 4 |  | 74 | 161 ± 15 |  | 74 | 295 ± 28 |  | 74 | 559 ± 50 |  | 74 | 0.4 |
|  | **Low** | 75 | 71 |  | 75 | 49 ± 3 |  | 71 | 152 ± 14 |  | 71 | 284 ± 27 |  | 69 | 538 ± 54 |  | 69 | 0.5 |
|  | **Medium** | 69 | 66 |  | 69 | 49 ± 4 |  | 66 | 147 ± 15 |  | 66 | 277 ± 36 |  | 62 | 550 ± 101 |  | 62 | 2.8 |
|  | **High** | 73 | 70 |  | 73 | 50 ± 3 |  | 70 | 146 ± 17 |  | 57 | 265 ± 41 |  | 47 | 459 ± 170 |  | 47 | 6.6 |
| **A** | **-** | 71 | 66 |  | 71 | 48 ± 5 |  | 66 | 155 ± 17 |  | 66 | 295 ± 31 |  | 66 | 542 ± 58 |  | 66 | 0.3 |
|  | **Low** | 73 | 62 |  | 73 | 48 ± 4 |  | 62 | 154 ± 17 |  | 58 | 277 ± 39 |  | 50 | 521 ± 77 |  | 50 | 2.5 |
|  | **Medium** | 72 | 68 |  | 72 | 48 ± 5 |  | 68 | 149 ± 18 |  | 59 | 256 ± 34 |  | 45 | 491 ± 111 |  | 45 | 4.1 |
|  | **High** | 75 | 70 |  | 75 | 48 ± 4 |  | 70 | 141 ± 17 |  | 33 | 222 ± 39 |  | 7 | 387 ± 116 |  | 7 | 10.3 |
| **B** | **-** | 72 | 64 |  | 72 | 46 ± 4 |  | 64 | 147 ± 18 |  | 64 | 266 ± 32 |  | 64 | 504 ± 58 |  | 64 | 0.4 |
|  | **Low** | 72 | 68 |  | 72 | 45 ± 5 |  | 68 | 145 ± 21 |  | 66 | 254 ± 42 |  | 61 | 469 ± 93 |  | 61 | 2.9 |
|  | **Medium** | 74 | 66 |  | 74 | 46 ± 5 |  | 66 | 140 ± 17 |  | 63 | 231 ± 34 |  | 49 | 428 ± 113 |  | 49 | 5.4 |
|  | **High** | 71 | 64 |  | 71 | 46 ± 4 |  | 64 | 142 ± 17 |  | 49 | 214 ± 41 |  | 34 | 371 ± 150 |  | 34 | 7.5 |

^1^*E. coli* was inoculated intratracheally on day 8. Low, medium and high inoculations dose were 10^5.1^, 10^6.1^, and 10^7.1^ colony forming units (CFU) per bird, respectively.

^2^Number of birds

^3^Body weight ± standard deviation
