# Supplementary material for: Quantitative assessment and comparison of susceptibility to colibacillosis in pure lines of broiler breeders and their commercial offspring
Source: Poult Sci. 2025 Aug 24;104(11):105722. doi: 10.1016/j.psj.2025.105722 (PMC12451321; doi:10.1016/j.psj.2025.105722)
Supplement: Supplementary file 7 [file mmc7.docx]

**Supplementary Table 3.** Overview of the results of experiment 3.

|  |  |  |  |  |  | Bodyweight at day (grams) | | | | | | | | | | | | | | | | | |  | Surving chickens at end of experiment | |  |
| --- | --- | --- | --- | --- | --- | --- | --- | --- | --- | --- | --- | --- | --- | --- | --- | --- | --- | --- | --- | --- | --- | --- | --- | --- | --- | --- | --- |
|  |  |  |  |  |  | 1 | |  | | 7 | | |  | | 15 | | |  | 21 | |  | 28 | |  |  |  |  |
| Chicken line | IBV  Inoculation^1^ | *E. coli* inoculation^2^ | Number of birds allocated to group | Number of Inoculated birds |  | N=^3^ | BW ± SD^4^ | |  | | N= | BW ± SD | |  | | N= | BW ± SD |  | N= | BW ± SD |  | N= | BW ± SD |  | N= | Mean lesions score | |
| **Commercial** | **-** | **-** | 30 | 20 |  | 30 | 54 ± 3 | |  | | 30 | 162 ± 8 | |  | | 30 | 366 ± 77 |  | 20 | 778 ± 40 |  | 20 | 1273 ± 62 |  | 20 | 0.1 | |
|  | **-** | **+** | 30 | 20 |  | 30 | 55 ± 4 | |  | | 30 | 177 ± 20 | |  | | 30 | 380 ± 41 |  | 20 | 774 ± 48 |  | 17 | 1177 ± 188 |  | 17 | 3.0 | |
|  | **+** | **+** | 30 | 20 |  | 30 | 54 ± 4 | |  | | 30 | 164 ± 13 | |  | | 30 | 387 ± 32 |  | 20 | 730 ± 28 |  | 17 | 986 ± 163 |  | 17 | 7.9 | |
| **A** | **-** | **-** | 30 | 20 |  | 30 | 50 ± 4 | |  | | 29 | 132 ± 27 | |  | | 25 | 400 ± 72 |  | 20 | 736 ± 74 |  | 20 | 1283 ± 118 |  | 20 | 0.3 | |
|  | **-** | **+** | 30 | 19 |  | 30 | 51 ± 4 | |  | | 29 | 145 ± 22 | |  | | 28 | 423 ± 74 |  | 19 | 777 ± 65 |  | 9 | 1094 ± 257 |  | 9 | 3.9 | |
|  | **+** | **+** | 30 | 20 |  | 30 | 52 ± 4 | |  | | 29 | 140 ± 21 | |  | | 29 | 376 ± 70 |  | 20 | 669 ± 62 |  | 8 | 795 ± 205 |  | 8 | 8.4 | |

^1^Infectious bronchitis virus, vaccine strain H52, was inoculated intratracheally and oculonasally on day 16 at a dose of 10^5.5^ EID50 per bird.

^2^*E. coli* inoculations were performed on day 21, with a dose of 10^7.0^ colony forming units per bird administered.

^3^Number of birds

^4^Body weight ± standard deviation
